# Editorial: Revisiting immunological roles for bone marrow stromal cell antigen-1; an entero-neuro-immune regulator

**DOI:** 10.3389/fimmu.2023.1239546

**Published:** 2023-06-27

**Authors:** Ada Funaro, Takashi Nakagawa, Katsuhiko Ishihara

**Affiliations:** ^1^ Department of Medical Sciences, University of Turin, Turin, Italy; ^2^ Department of Molecular and Medical Pharmacology, Faculty of Medicine, University of Toyama, Toyama, Japan; ^3^ Department of Design for Medical and Health Care, Faculty of Health and Welfare Services Administration, Kawasaki University of Medical Welfare, Okayama, Japan

**Keywords:** BST-1/CD157, ADP-ribosyl cyclases, NAD+, immune regulation, social behavior, neuropsychiatric diseases

BST-1/CD157 is a member of the ADP-ribosyl cyclases (ARC) gene family, that metabolizes NAD+ and exerts receptor functions (1, 2). Originally identified as bone marrow stromal (BST) and myeloid cell differentiation antigen, CD157 turned out to have a wider expression than originally assumed and to be involved in a number of physiological and pathological contexts. Thus, after a long time in the shadow of its paralogue CD38, the second member of the ARC family, CD157 has seen a renaissance. CD157’s functions were found to go well beyond its roles in immune responses. Indeed, in addition to being a key regulator of leukocyte adhesion, migration and diapedesis (3, 4) and of innate immunity (5, 6), it also emerged as neuroregulator affecting emotions and social behavior (7–9) and as a marker of endothelial stem cells (10) and regulator of hemopoietic, mesenchymal (11) and intestinal stem cells (12).

Recently, new insights concerning the enzymatic activity highlighted that CD157 (but not CD38) has both hydrolase activity and base-exchange activity specific to the NAD^+^ precursor nucleoside nicotinamide riboside (NR), suggesting that CD38 and CD157 evolved toward distinct substrate preferences (13).

In pathological conditions, CD157 has been reported to promote disease progression in epithelial ovarian cancer and malignant pleural mesothelioma (14, 15). In acute myeloid leukemia CD157-driven intracellular signals protect leukemic cells from apoptosis and dampen their sensitivity to chemotherapy (16). Although its pathophysiological significance in the central nervous system is still unclear, clinical studies revealed relationships between *BST-1/CD157* genetic variants and neuropsychiatric diseases, including Parkinson’s disease (PD) (17), autism spectrum disorders (18), sleep disorders (19), depressive disorders and restless leg syndrome (20).

This Research Topic brings together five papers -briefly described below- that are intended to provide an overview about the multifaceted roles of BST-1/CD157 not limited to immunity.

Lopatina et al., highlighted the complex role of CD157 at the crossroad of neurogenesis, cerebral angiogenesis, and immune regulation, proposing that CD157 might serve as an important component of innate immune reactions in the central nervous system. Considering the well-established role of CD157 in the regulation of leukocytes trafficking and its cross-talk with selected integrins, the authors envision that integrin-mediated control of the blood-brain barrier (BBB) integrity might be partly provided by CD157 expressed on endothelial cells lining the brain microvessels. In addition, CD157 expressed by neutrophils and endothelial cells could contribute to the pathogenesis of immune response-associated BBB breakdown and progression of neuroinflammation, due to excessive migration of leukocytes from the blood into the brain at the sites of compromised BBB permeability. Comparison of CD157KO and wild-type C57BL/6 mice, demonstrated that loss of CD157 suppresses synaptogenesis in the hippocampus, whereas CD157 overexpression in brain cells is paralleled by progression of neurodegeneration.

Overall, the authors conclude that CD157 could control structural and functional integrity of the BBB and barrier genesis thus influencing brain plasticity.

Xia et al. characterized thymic portal endothelial cells (TPEC) as the cellular basis of the thymic homing hematopoietic progenitor cells (HPC). The authors identified of two functional TPEC subsets (namely, C2 and C6) with different BST-1/CD157 expression levels: BST-1^hi^ or BST-1^lo/-^. Notably, mature thymocytes exit takes place at BST-1^hi^ vessels, while HPCs entry takes place at BST-1^lo/−^ vessels; hinting that, in the mouse, BST-1/CD157 is an appropriate marker for discriminating entry and exit site of thymus, although it apparently does not regulate thymic egress. The definition of these BST-1^hi^ or BST-1^lo/−^ subsets may shed new light to the mechanistic study of thymic trafficking and highlights a role of BST-1/CD157 in the thymocyte-thymic stroma cross-talk.

Higashida et al., review the role of CD38, CD157, and receptor for advanced glycation end-products (RAGE) in the control of social memory and behavior in mice through the regulation of the oxytocin (OT) concentrations in the brain and blood. Briefly, RAGE regulates OT dynamics at the interface among the brain, blood, and intestine, *in vivo*. Indeed, extracellular OT concentrations in the brain could be mediated by the unidirectional transport of OT by RAGE from the blood to the brain and release of OT from oxytocinergic neurons by CD38 and CD157. Previous evidence indicated that in the hypothalamus, cADPR produced by CD38 and (to a lesser extent) by CD157 through their catalytic activity, increases intracellular free Ca^2+^ concentrations causing OT release from oxytocinergic neurons. Disruption of social memory and recognition or parental nurturing behavior due to reduced OT release in CD38KO mice, support this notion.

Oxytocin is the common link between this review and the research article by Gerasimenko and Higashida addressing the roles of CD38 and CD157 in social behavior. Starting from their previous observation that cADPR, a Ca^2+^ mobilizing second messenger derived by CD38- and CD157-mediated NAD^+^ metabolism, is critical in releasing OT from the hypothalamus into the brain, the authors investigated whether nicotinamide riboside (NR, an orally active NAD^+^ precursor) could mitigate the social behavior defects in CD38KO and CD157KO mice. Gavage administration of NR for 12 days was safe and had no significant effects on mice sociability, while it ameliorated social preference defects in CD157KO but not in CD38KO mice. The authors attribute the different effect of NR in the two KO models sharing a similar phenotype, to a drastic reduction of cADPR in CD38KO but not in CD157KO mice, which differently affects the oxytocin release. Different levels of oxytocin affect sociability, social preference, and social memory to variable extents. Hence, oral administration of NR proved sufficient to recover social preference defects in CD157KO by increasing oxytocin release. Noteworthy, in the small intestine and liver, CD157 specifically degrades NR, while CD38 does not (13), hinting to the existence of tissue-specific metabolic pathways of orally administered NR. This is an intriguing question that warrant further investigation. As NR exhibits beneficial effects in preventing aging-related physical decline and diseases (21), it will be of tremendous interest to determine whether CD157 is involved in aging and inflammation through the degradation of NR.

In the past decade, a number of studies have suggested that the *BST-1/CD157* gene could be a risk locus for several neuropsychiatric disorders. Yokoyama summarizes the structure of the *BST-1/CD157* gene, its expression in the nervous system and its relationships with neuropsychiatric disorders. Studies performed in populations with different ethnic backgrounds have identified nearly ten PD-associated intronic SNPs, for some of which the association was statistically significant in some populations but not in others. These SNPs represent common variants with a frequency >10% in the population, indicating that none of them could be an appropriate predictive biomarker for disease susceptibility. However, whether these SNPs could be integrated into polygenic risk score analysis in combination with other PD-related genes remains to be defined. Three SNPs (rs4301112, rs28532698, and rs10001565) different from PD-associated ones, were found associated with autism spectrum disorders, one of which also showed association with major depressive disorder, according to the notion that individual SNPs are often associated with multiple forms of psychopathology. The fil rouge among these diseases is anxiety and depression, both behavioral defects recapitulated in CD157KO mice. Functional genomic analyses are needed to determine which variants may hold biological relevance.

This Research Topic of Frontiers in Immunology will hopefully provide the reader with an overview of the role of BST-1/CD157 NAD+ metabolizing ectoenzyme in multiple physiological and pathological contexts emphasizing light and shadow. In recent years, burgeoning research on CD157 has broadened the horizon far beyond its role as an immunoregulator (22), raising new challenges. Among these, to understand the role of CD157 in the gut-brain axis influencing a variety of brain disorders is a top priority.

## Author contributions

All authors listed have made a substantial, direct, and intellectual contribution to the work and approved it for publication.
